# Immune Response to Hepatitis B Virus Vaccine Among People Living With HIV: A Meta-Analysis

**DOI:** 10.3389/fimmu.2021.745541

**Published:** 2021-12-22

**Authors:** Yakun Tian, Wei Hua, Yaxin Wu, Tong Zhang, Wen Wang, Hao Wu, Caiping Guo, Xiaojie Huang

**Affiliations:** Center for Infectious Diseases, Beijing Youan Hospital, Capital Medical University, Beijing, China

**Keywords:** human immunodeficiency virus, hepatitis B virus, vaccine, immune response, response rate

## Abstract

**Background:**

There is conflicting evidence about whether a double dose of the hepatitis B virus (HBV) vaccine induces better immunity than the standard-dose vaccine for people living with HIV (PLWH). This study provides a meta-analysis that summarizes the efficacy of HBV vaccine regimens among HIV-infected patients, clarifying the role of particular factors such as dose and frequency of vaccination in vaccine responsiveness and highlighting the need for evidence-based practice to assess HBV vaccination among PLWH.

**Methods:**

Randomized clinical trials (RCTs) and prospective studies reporting vaccination response rates among PLWH were found through a search of PubMed, Cochrane, and the Web of Science. The key outcome was vaccine response. A random-effects model was used to estimate the pooled response rate. Subgroup analysis was conducted to evaluate key factors and explore sources of heterogeneity. Possible biases were assessed using quality and publication bias assessment.

**Results:**

Eligible studies included controlled trials that examined the effects of 17 interventional studies with 1,821 participants. Among PLWH who received the HBV vaccine, the pooled response rate of HBV vaccination was 71.5% (95% CI 64.0%–77.9%, *p* < 0.001). Compared with the standard dose (65.5%, 95% CI 53.1%–76.1%), the double dose (75.2%, 95% CI 66.2%–82.5%) was associated with a better response rate [*Q*(1) = 19.617, *p* < 0.001]. When stratified by schedule, the four-dose schedule (89.7%, 95% CI 83.1%–93.9%) had a higher response rate than the three-dose schedule (63.3%, 95% CI 56.6%–69.4%) and the difference was significant [*Q*(1) = 88.305, *p* < 0.001]. PLWH with higher CD4^+^ T-cell counts (>500 cells/mm^3^) at the time of vaccination had better response rates [*Q*(1) = 88.305, *p* < 0.001].

**Conclusions:**

In this meta-analysis, the double dose of the HBV vaccine and multiple injections were associated with better immune responses than the standard HBV vaccine regimen in PLWH. Higher seroconversion rates were observed in PLWH with high CD4^+^ T-cell levels, indicating that individuals infected with HIV should receive the HBV vaccine as soon as possible after diagnosis.

**Systematic Review Registration:**

https://www.crd.york.ac.uk/PROSPERO/.

## Introduction

Given that human immunodeficiency virus (HIV) and hepatitis B virus (HBV) share similar routes of transmission, coinfection with the two viruses is common (1). Globally, almost 10% of people living with HIV (PLWH) are infected with HBV (2, 3). HBV-related liver disease affects the life expectancy of PLWH. In individuals infected with HIV, HBV more often becomes a chronic infection, with patients more likely to experience progression to cirrhosis, hepatocellular carcinoma (4, 5), and liver failure (6, 7). While antiretroviral therapy (ART) has led to a decline in AIDS-related mortality, liver disease remains a major cause of morbidity and mortality in PLWH (8–10).

International guidelines highly recommend HBV immunization (11, 12). HBV infection has declined with the popularization of HBV vaccines, especially as current A2 recombinant HBV vaccines show cross-reactivity and cross-protection against the non-A2 HBV genotype (13). U.S. and British guidelines specify that PLWH should also be vaccinated for HBV. HBV vaccine responsiveness is measured by assessing seroconversion after vaccination, with HBV surface antibody [anti-HBs] ≥10 IU/L measured at 4–8 weeks after the last dose defined as a positive response. While the HBV vaccine has been certified by the CDC as an effective way to prevent HBV infection (14), PLWH often have a lower seroresponse after HBV vaccination than HIV-negative individuals. Indeed, immune responses to most vaccines are impaired in PLWH (15, 16). Several studies have worked on improving the response rate by changing the inoculation time and dose of the vaccine. While there is no consensus on an appropriate HBV vaccination schedule for PLWH, high-dose vaccine schedules may induce stronger response rates against HBV (17–20).

Several questions remain about the appropriate HBV vaccine schedule and dose for PLWH. This is in part because the number of PLWH who are susceptible to vaccine-preventable infectious diseases remains unknown and historic data from the pre-ART era may skew findings. Effective ART can increase CD4^+^ T-cell counts in PLWH, which strengthens both humoral and cellular immunity. The meta-analysis presented here summarizes the efficacy of HBV vaccines among PLWH. A subgroup analysis is conducted based on PLWH vaccination and treatment background, clarifying response rates and associated factors of the HBV vaccine among PLWH and providing evidence-based prevention recommendations.

## Methods

This review was registered in the International Prospective Register of Systematic Reviews (PROSPERO, CRD42018081009) and is reported fully in line with the Preferred Reporting Items for Systematic Reviews and Meta-analysis.

### Data Sources and Search Strategy

A comprehensive search of articles published in PubMed, Cochrane, and the Web of Science during January 2000 to April 2021 was conducted. Additional searches were conducted in Google Scholar and ClinicalTrials.gov. Keywords represented the intersection of HIV-related terms (HIV OR AIDS) and HBV-related terms (HBV vaccine OR vaccination). The reference lists of the included studies were also screened to ensure that no studies were omitted. Only peer-reviewed articles written in English were included.

### Study Selection and Data Extraction

Search results were initially imported into Endnote X8 to exclude duplicates. All titles and abstracts were screened by two authors to narrow the scope. Two investigators selected the remaining articles independently by full-text assessment if they 1) included adult HIV-positive patients, 2) evaluated the efficacy of the HBV vaccine, 3) provided vaccination at doses of 20 or 40 μg, and 4) provided sufficient data to calculate the effect size. Studies were excluded if 1) patients were positive for one of the HBV serological markers, 2) they were retrospective studies or case reports, and 3) they were not written in the English language.

Two authors independently extracted information using an Excel spreadsheet. The key outcome was vaccine response. Other information, such as study design, year of publication, author, sample size, sex distribution, CD4^+^ T-cell count schedule, and dose of HBV vaccine, was also extracted from the articles. Disagreements during the process were solved through negotiation.

Frequency of vaccination and dose of vaccination were defined to avoid ambiguous discussion. The “three-dose schedule” was defined as three intramuscular injections of HBV vaccine at months 0, 1, and 2 (or 6). The “four-dose schedule” was defined as four intramuscular injections at months 0, 1, 2, and 3 (or 6). Besides, standard dose (20 μg) and double dose (40 μg) were involved.

### Statistical Analysis

Comprehensive Meta-Analysis (CMA) Version 2.0 was used to conduct single rate and subgroup analysis. Combined event rate (ER) was calculated to measure the vaccine response rate among PLWH. A random-effects model was adopted using the ER to estimate the vaccine response. The *I* ^2^ statistic was used to assess heterogeneity across studies. Potential publication bias across included studies was measured using the Egger’s regression test and adjusted with trim-and-fill. For categorical moderators, subgroup analysis was also performed according to the HBV vaccine schedule (three-dose or four-dose), dose (standard dose or double dose), and CD4^+^ T-cell count (<500 or ≥500 cells/mm^3^). *p <*0.05 was considered statistically significant.

### Quality Assessment of Individual Studies

As there are prospective cohort and RCT studies included, we referred to the Newcastle-Ottawa quality assessment scale (NOS) and Cochrane Collaboration’s tool. The NOS evaluated the quality of prospective cohort studies with eight items from three areas: sample selection, comparability of cohorts, and outcome assessment. RCT studies were assessed for selection bias, performance bias, detection bias, attrition bias, reporting bias, and other biases.

## Results

### Characteristics of the Included Studies

**Figure 1:**
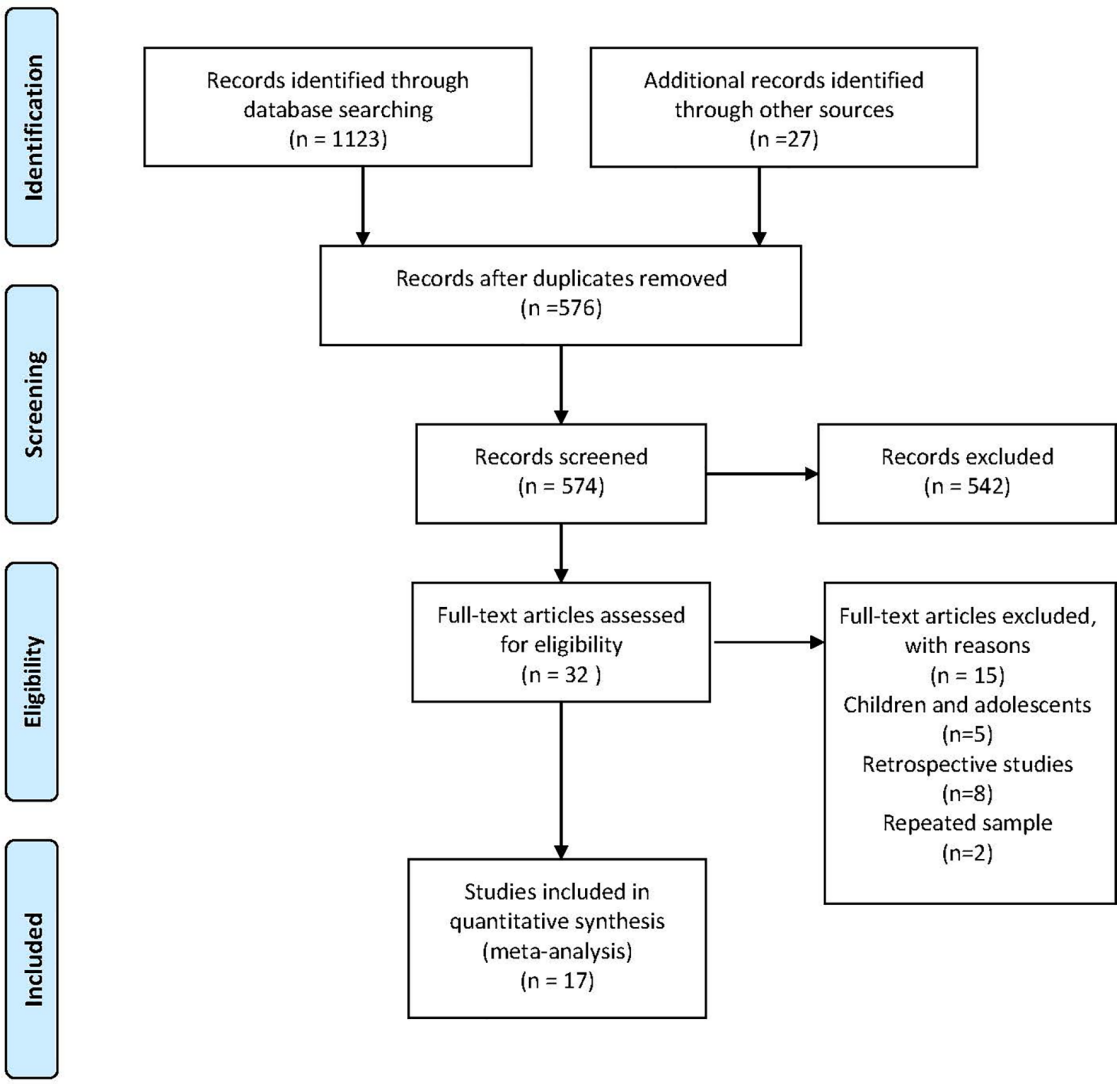
Flowchart of the study selection process.

Our search yielded 1,123 studies and 27 were identified by checking references and conferences. After discarding duplicates and screening the titles and abstracts, 32 articles remained for full-text assessment. Seventeen eligible studies consisting of 1,821 participants, with sample sizes ranging from 20 to 286, were included in the meta-analysis. The flow diagram of study selection is shown in **Figure 1**. The age of the patients ranged from 18 to 77 years old, and approximately 36.2% were female. **Table 1** summarizes the characteristics of the included studies which varied in study design, schedule, dose, comparison, CD4^+^ T-cell count, and HBV vaccine regimen. Seven studies were RCTs (17, 18, 20, 21, 26, 27, 32), while the remaining were prospective observational studies (22–25, 28–31, 33, 34). More than 80% (and sometimes even 100%) of the participants were on ART in the included studies. Of the 4 four-dose studies, all participants received the HBV vaccine at months 0, 1, 2, and 6 (20, 21, 33, 34). Unlike the four-dose regimen studies, the three-dose regimen studies differed in the time interval of the three injections. Eleven included studies with participants receiving the M0–1–6 regimen, three included studies with patients receiving the M0–1–2 regimen (22, 27, 31), and one included patients receiving the M0–1–3 regimen (32).

**Table 1:** Characteristics of the included studies.

Study	Year	Design	Sample size	Schedule	Response rate	*N*% on ART	Mean CD4 count (cells/μl)
**Fonseca et al.** (18)	2005	RCT	192	a. 20 μg M0–1–6 b. 40 μg M0–1–6	a. 34.0% b. 46.9%	a+b. 86%	429
**Launay et al.** (20)	2011	RCT	286	a. 20 μg M0–1–6 b. 40 μg M0–1–2–6	a. 64.5% b. 82.1%	a. 86% b. 80%	a. 516 b. 509
**Chaiklang et al.** (21)	2013	RCT	132	a. 20 μg M0–1–6 b. 20 μg M0–1–2–6 c. 40 μg M0–1–2–6	a. 86.6% b. 93.2% c. 95.5%	a. 100% b. 100% c. 100%	a. 400 b. 544 c. 544
**David Rey et al.** (17)	2015	RCT	178	a. 20 μg M0–1–6 b. 40 μg M0–1–6	a. 67% b. 74%	a. 87% b. 86%	a. 254 b. 207
**Rey et al.** (22)	2000	Prospective	20	20 μg M0–1–2	55%	85%	470
**Paitoonpong et al.** (23)	2008	Prospective	28	20 μg M0–1–6	71.4%	100%	324
**Ungulkraiwit et al.** (24)	2007	Prospective	65	20 μg M0–1–6	46.2%	88%	345
**Fuster et al.** (25)	2016	Prospective	245	20 μg M0–1–6	62%	94.7%	406
**Sasaki Md (** 26 **).**	2003	RCT	40	40 μg M0–1–6	60%	99%	462
**Cooper et al.** (27)	2005	RCT	19	40 μg M0–1–2	89%	100%	NA
**Pasricha et al.** (28)	2006	Prospective	40	NA	82.5%	0	NA
**Viega et al.** (29)	2006	Prospective	47	NA	63.8%	91%	NA
**Cornejo-Juarez et al.** (30)	2006	Prospective	40	40 μg M0–1–6	60%	65%	225
**Cruciani et al.** (31)	2009	Prospective	65	40 μg M0–1–2	60%	80%	533
**Overton et al.** (32)	2010	RCT	23	40 μg M0–1–3	65.2%	77%	446
**Potsch et al.** (33)	2010	Prospective	47	40 μg M0–1–2–6	89%	79%	402
**Potsch et al.** (34)	2012	Prospective	163	40 μg M0–1–2–6	91%	80%	NA

ART, antiretroviral therapy; RCT, randomized controlled trial; M, month; NA, not assessed.

Three RCTs showed high risk in the process of random sequence generation and two showed attrition bias and reporting bias, respectively. Two cohort studies included different populations in the two groups and one study did not assess potential confounding factors. In addition, four prospective cohort studies were single-arm studies and lacked information about non-exposed patients and comparability. Details of quality assessment of individual studies are shown in **Supplementary Tables S1, S2**.

### Vaccine Response

**Figure 2:**
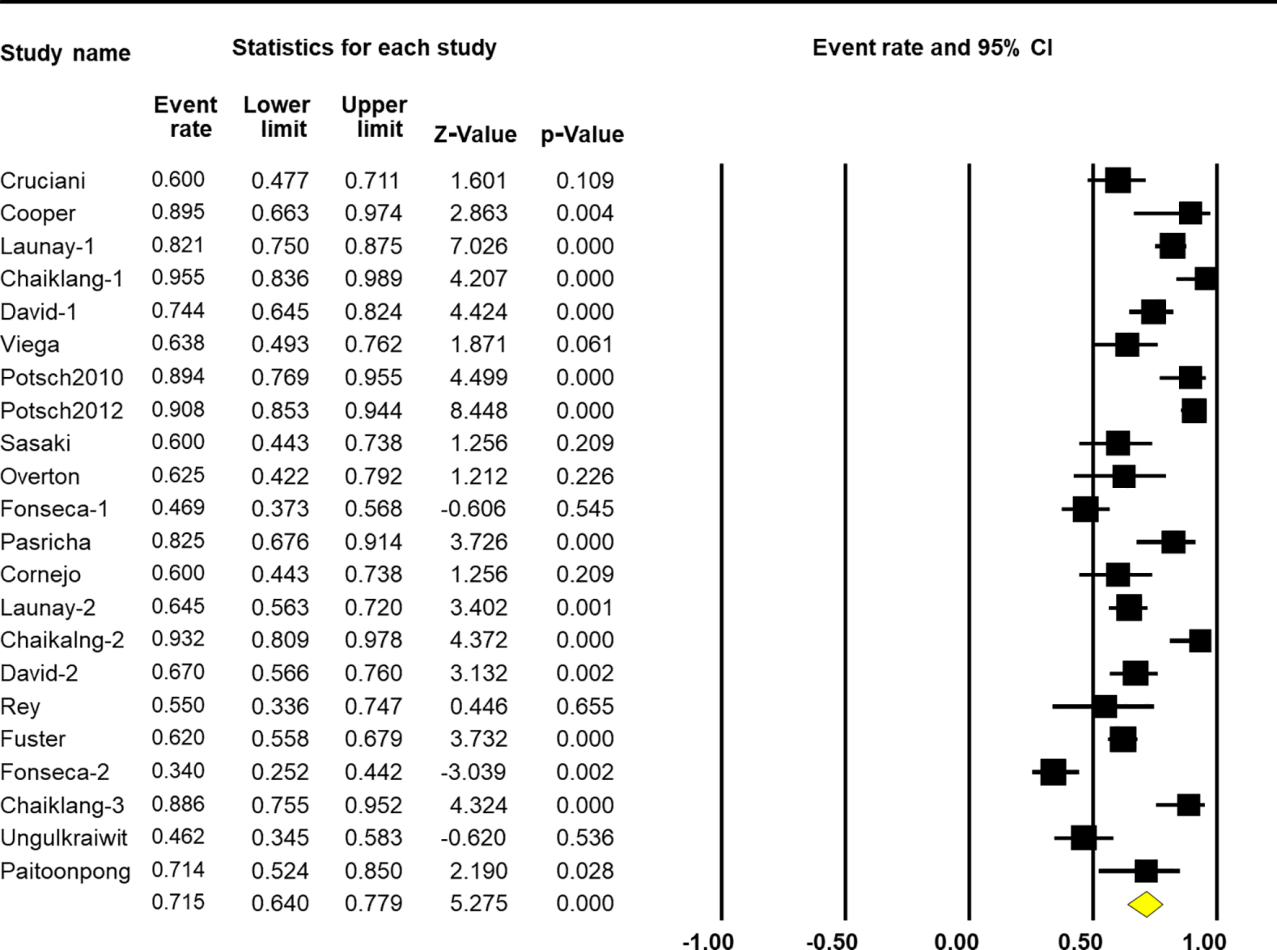
Overall efficacy of hepatitis B virus (HBV) vaccination on the response rate.

**Figure 3:**
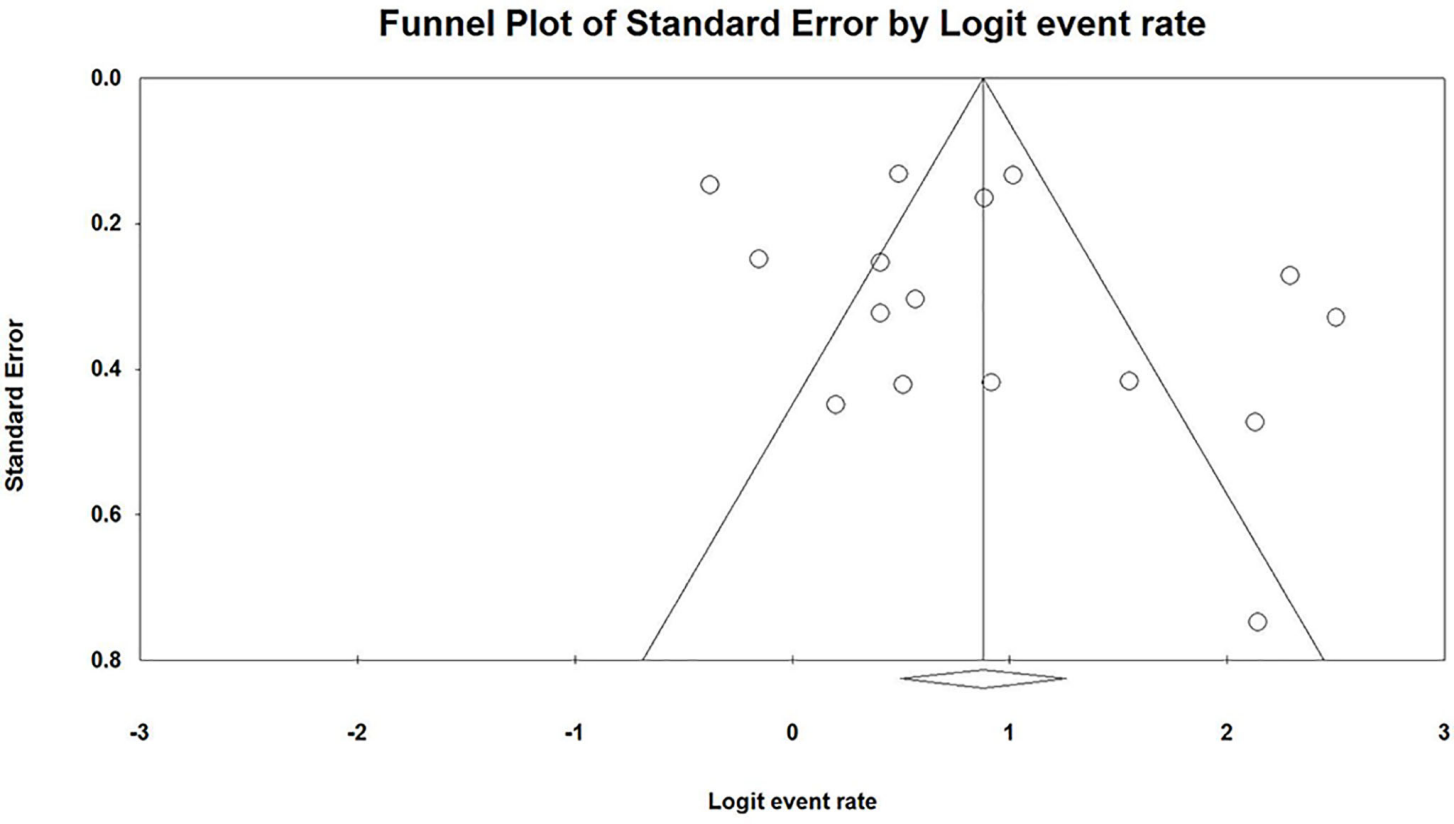
Funnel plot of all included studies.

The pooled response rate of the HBV vaccine was 71.5% (95% CI 64.0%–77.9%, *p* < 0.001, **Figure 2**). However, there was significant heterogeneity across individual studies [*Q*(22) = 165.005, *p* < 0.001, *I* ^2^ = 86.7%]. Egger’s regression showed no publication bias across studies with *p*-values of 0.096 and 0.191 in one-tailed and two-tailed analyses, respectively. The funnel plots are shown in **Figure 3**.

### Factors Associated With Response Rate

**Figure 4:**
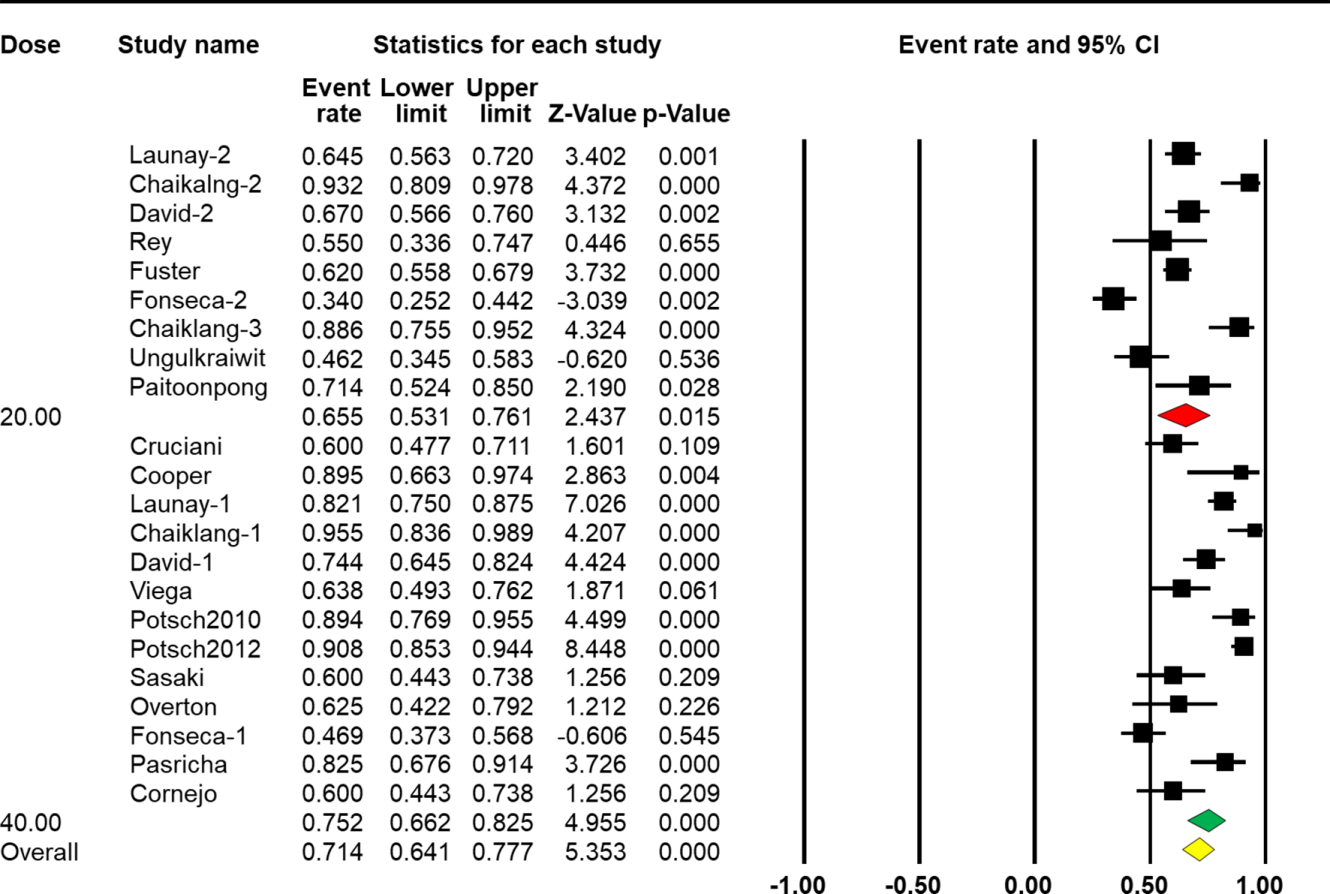
Efficacy of HBV vaccination at different doses (standard dose vs. double dose) on the response rate.

When stratified by vaccine dose, results from the subgroup analysis showed a significant difference across groups [*Q*(1) = 19.617, *p* < 0.001, **Table 2**]. Nine arms reported the effect of HBV vaccination at a standard dose (20 μg), so the combined ER of the vaccine response rate was 65.5% (95% CI 53.1%–76.1%, *p* = 0.015, **Figure 4**). Thirteen arms reported the effect of HBV vaccination at a double dose (40 μg), so the combined ER of the response rate was 75.2% (95% CI 66.2%–82.5%, *p* < 0.001, **Figure 4**).

**Table 2:** Meta-analysis results assessing the efficacy of HBV vaccination in response rates across subgroups.

Variables	Level	No. of comparisons	Event rate (95% CI)	*I* ^2^	Test for between-group homogeneity		
					*Q*	*df*	*p*-value
Schedule					88.305	1	0.000
	Three-dose	17	63.3 (56.6–69.4)	78.32			
	Four-dose	5	89.7 (83.1–93.9)	56.32			
Dose					19.617	1	0.000
	Standard	9	65.5 (53.1–76.1)	86.65			
	Double	13	75.2 (66.2–82.5)	86.92			
CD4 counts (cells/μl)					16.874	1	0.000
	<500	14	67.1 (56.4–76.3)	89.06			
	≥500	8	77.6 (68.4–84.7)	80.33			

**Figure 5:**
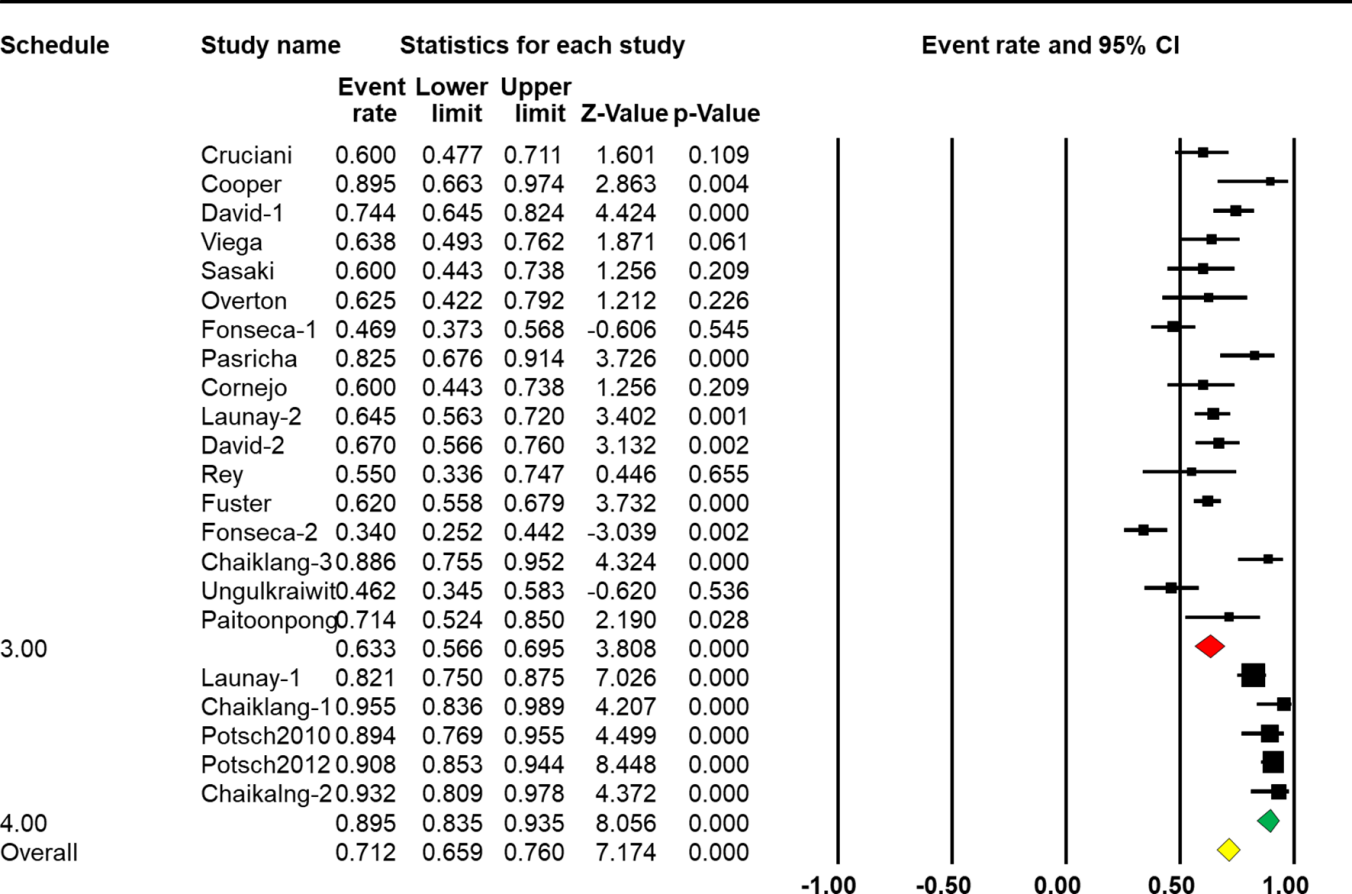
Efficacy of HBV vaccination with different schedules (three-dose vs. four-dose) on the response rate.

The four-dose schedule showed a higher response rate than the three-dose schedule [*Q*(1) = 88.305, *p* < 0.001, **Table 2**]. Seventeen arms reported the effect of HBV vaccination with three intramuscular injections, and the combined vaccine rate was 63.3% (95% CI 56.6%–69.4%, *p* < 0.001, **Figure 5**). From five arms, the combined response rate of HBV vaccination with four intramuscular injections was 89.7% (95% CI 83.1%–93.9%, *p* < 0.001, **Figure 5**).

**Figure 6:**
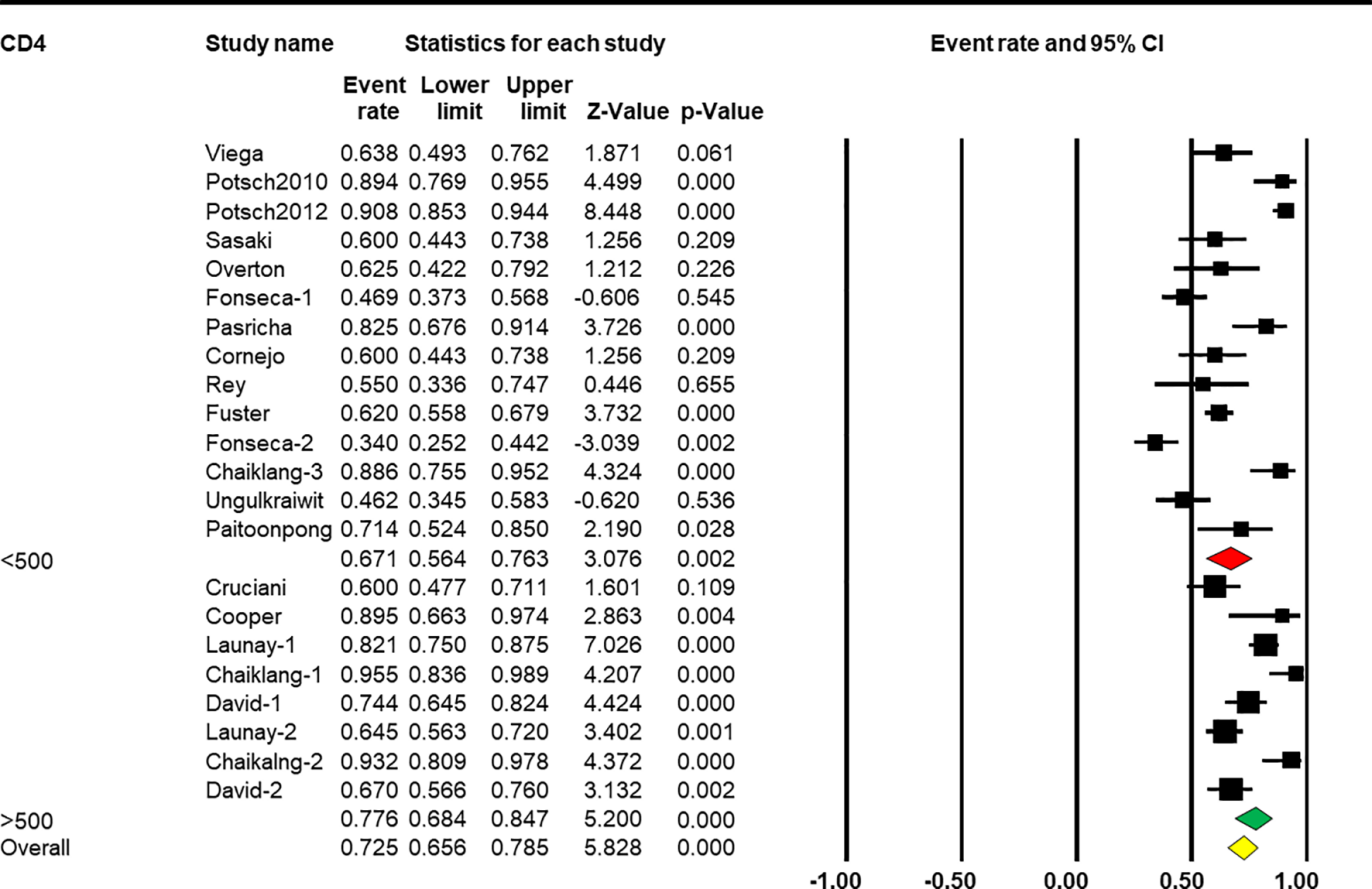
Efficacy of HBV vaccination with different CD4 stratification (<500 vs. ≥500 cells/mm^3^) on the response rate.

Subgroup analysis based on CD4^+^ T-cell count showed that a higher CD4^+^ T-cell count was associated with a better response rate [*Q*(1) = 88.305, *p* < 0.001, **Table 2**]. Eight arms reported the effect of HBV vaccination with more than 500 cells/mm^3^ and the combined ER of the response rate was 77.6% (95% CI 68.4%–84.7%, *p* < 0.001, **Figure 6**). Fourteen arms reported the effect of HBV vaccination with less than 500 cells/mm^3^ and the combined ER of the response rate was 67.1% (95% CI 56.4%–76.3%, *p* = 0.002, **Figure 6**).

## Discussion

The aim of this meta-analysis was to pool available data on the response rates of HBV vaccines in PLWH. To provide credible evidence, only RCT and prospective studies were included. The findings indicated that both a double dose of the HBV vaccine and the four-dose schedule were associated with a better immune response than the standard HBV vaccine regimen in PLWH. In addition, higher seroconversion rates were observed in PLWH with CD4^+^ T-cell levels >500 cells/mm^3^.

By analyzing 17 studies that met the inclusion criteria during 2000 to 2016, the pooled response rate of HBV vaccination for PLWH was 71.5% (95% CI 64.0%–77.9%, *p* < 0.001, **Figure 2**). Using the “classic schedule” (20 μg of HBsAg at months 0–1–6), PLWH response rates to the vaccine were lower than those of non-HIV-infected people (20%–70% vs. 90%–95%) (22, 35–38). According to a previous study, high plasma viral load, prevaccination total serum IgG, and elevated prevaccination IgG1 among PLWH were associated with poor vaccine response (38).

The double-dose HBV vaccine regimen had a significantly higher response rate than the standard-dose regimen (75.2% and 65.5%, respectively; *p* < 0.001). Several studies (20, 33, 34, 39) have shown that PLWH who have never received the HBV vaccine had higher response rates after the double-dose vaccination. Moreover, two meta-analyses found that a higher dose vaccination could improve the anti-HB immune reaction in previously unvaccinated patients and could ultimately result in a high response rate (OR 1.96, 95% CI 1.47–2.61) (19, 40). Beginning in 2013, the British HIV Association recommended 40-μg doses of HBV vaccine for primary vaccination and revaccination of PLWH (41). In some studies, double-dose groups had higher and more long-lived anti-HB titers, which warrants additional study. However, other studies indicated that there is no relationship between vaccine dose and level of anti-HB response (17, 18, 21). In an open-label RCT, a total of 178 PLWH were randomized to receive either standard-dose (20 mg, three-dose) or double-dose (40 mg, three-dose) HBV vaccines at months 0, 1, and 6. A similar response was seen between the standard-dose and double-dose groups (67% vs. 74%, *p* > 0.05) (17). Another RCT by Fonseca et al. reported that seroconversion in response to 20 and 40 mg of the three-dose regimen among PLWH were 34% and 47%, respectively, at week 28. However, this difference was not significant (*p* > 0.05) (18).

In addition to double dose, several studies have shown that increased HBV vaccine schedules are more effective in PLWH. Launay et al. (20) proved that PLWH vaccinated with a four-double-dose regimen had higher anti-HBV titers and stronger immune responses than those vaccinated with the standard three-dose regimen (82% vs. 65%, *p* < 0.05). Chaiklang et al. compared the immunogenicity and safety of three-standard dose and four-double dose vs. four-standard dose in an RCT (21). Higher response rates were not observed in the double-dose or four-double-dose groups. This may have been impacted by low CD4^+^ T cell counts, high viral load of the participants, and small sample size. The meta-analysis described here showed a similar result. However, the subgroup analysis did not include the time interval of the three-dose regimen studies because the number of studies including three subgroups varied. Further studies are needed to explore the relationship between immune response and time interval for three- or four-dose HBV vaccination regimens in PLWH.

Several studies have found that the response to HBV vaccination is significantly associated with undetectable virus load (29, 42) and high or even close to normal CD4^+^ T-cell counts (18, 22, 29) in PLWH. Veiga et al. (29) found that vaccine responders had higher CD4^+^ T-cell numbers than non-responders (452 vs. 359 cells/mm^3^). The results shown in this meta-analysis were similar. PLWH with higher CD4^+^ T-cell counts had a stronger response to HBV vaccination than those with low CD4^+^ T-cell counts, especially those with a baseline CD4^+^ T-cell count >500 cells/mm^3^ (*p* < 0.001). It is suggested that vaccination as early as possible after diagnosis with high CD4^+^ T-cell counts is necessary for PLWH. However, according to the CDC, vaccination should not be deferred until CD4^+^ T-cell counts return to a certain level because patients with low CD4^+^ T-cell counts can still mount a response to the HBV vaccine (43). The lack of response to the HBV vaccine is attributed to a variety of immunological mechanisms, including antigen presentation of the peptide-based vaccine to T cells as well as B-cell activity. The functions of T and B cells in HBV vaccine non-responders are complicated. S gene escape mutants created after HBV vaccination may change host susceptibility or lead to vaccine escape from immune attack, as shown previously (44).

Immune responses are lower following HBV vaccination of PLWH than individuals without HIV infection. For PLWH, achieving a durable and protective level of immunity remains a challenge, especially for those with detectable HIV RNA or low CD4^+^ T-cell counts at the time of vaccination. For multiple reasons, completing and maintaining adequate HBV immunity in HIV-infected individuals is complex, and new strategies have emerged to overcome these barriers (45). Some specialists suggest waiting for HIV viral suppression and immune reconstitution before vaccination (30, 33). One study recommended a second vaccine series for PLWH with high CD4^+^ T-cell counts and undetectable HIV RNA who do not respond to HBV vaccination (17). The benefits of double dosage and increased dose frequency remain controversial. In this meta-analysis, results indicated that a higher vaccine dose, higher CD4^+^ T-cell levels, and multiple injections are associated with a better response to HBV vaccination in PLWH. In patients with lower CD4^+^ T-cell counts, higher HBV vaccine doses or increased dose frequency may be warranted to accentuate the immune response, and in patients with higher CD4^+^ T-cell counts, standard HBV vaccination should be performed as soon as possible after diagnosis. Importantly, the role of ART in vaccination is not evaluated in our study and there is only one study conducted in treatment-naive PLWH (28). Data showed that even after a double dose of vaccine, HBsAb titers were much lower in HIV-positive patients than HIV-negative adults.

This study has some limitations. The HIV patients included were from many different regions, including the Americas (USA 2, Brazil 5, Mexico 1, and Chile 1), Europe (France 3 and Italy 1), and East Asia and Southeast Asia (India 1 and Thailand 3). However, studies from the country with the highest HBsAg prevalence, Africa (46), are absent. The heterogeneity of this analysis was significant (*I* ^2^ > 50%) and the observed differences may be due to differences in sample size, demographics, and CD4^+^ T-cell counts or inadequate statistical power. The results of this study are credible but need to be interpreted with caution.

Besides, the funnel plot showed that some studies are outside the scope of the confidence interval (triangle jurisdiction). The scattered funnel plot also revealed significant heterogeneity in our study. However, the random-effect model and the performed subgroup analysis were adopted to reduce the effect of heterogeneity. The asymmetric funnel plot also suggested that there was possible publication bias; however, we conducted a comprehensive search to make sure no study was omitted.

## Conclusion

PLWH have significantly lower initial response rates after HBV vaccination than immunocompetent individuals. Additional vaccine doses and other methods for strengthening immunity should be considered for PLWH. This meta-analysis indicated that there were higher seroprotection rates to HBV vaccine in response to double-dose vaccination, increased dose frequency, and higher baseline CD4^+^ T-cell counts. To obtain the best response to hepatitis B vaccine in PLWH, additional large-scale studies that explore the role of other potential factors should be conducted in the future.

## Data Availability Statement

The raw data supporting the conclusions of this article will be made available by the authors, without undue reservation.

## Author Contributions

XH, CG, and HW conceptualized the study and developed the research protocol. YT, WH, and YW identified articles for full-text review and extracted data that matched the inclusion criteria. YT performed the statistical analyses. All authors contributed to the writing of the manuscript. XH, TZ, and WW polished and revised the manuscript. All authors contributed to the article and approved the submitted version.

## Funding

This work was supported by the National Science and Technology Major Project of China During the 13th Five-year Plan Period (2017ZX10201101, 2018ZX10715-005-002-002), the Beijing Excellent Talent Plan (2018000021223ZK04), the Beijing Talent Project in the New Millennium (2020A35), and Beijing Hospitals Authority “Peak Climbing” Planning (DFL20191701).

## Conflict of Interest

The authors declare that the research was conducted in the absence of any commercial or financial relationships that could be construed as a potential conflict of interest.

## Publisher’s Note

All claims expressed in this article are solely those of the authors and do not necessarily represent those of their affiliated organizations, or those of the publisher, the editors and the reviewers. Any product that may be evaluated in this article, or claim that may be made by its manufacturer, is not guaranteed or endorsed by the publisher.
