# Supplementary material for: Immune Response to Hepatitis B Virus Vaccine Among People Living With HIV: A Meta-Analysis
Source: Front Immunol. 2021 Dec 22;12:745541. doi: 10.3389/fimmu.2021.745541 (PMC8728056; doi:10.3389/fimmu.2021.745541)
Supplement: Supplementary file 1 [file Table_1.docx]

Supplementary Material

# Supplementary Figures and Tables

## Supplementary Tables

Table S1. Quality assessment of RCT

RCT: Randomized Controlled Trial

| **Author** | **year** | **Selection bias** | | **Performance bias** | **Detection bias** | **Attrition bias** | **Reporting bias** | **Other bias** |
| --- | --- | --- | --- | --- | --- | --- | --- | --- |
|  |  | **Random sequence generation** | **Allocation concealment** | **Blinding of participants and personnel** | **Blinding of outcome assessment** | **Incomplete outcome data** | **Selective reporting** | **Other sources of bias** |
| Sasaki Md. | 2003 | High risk | Low risk | Low risk | Low risk | Low risk | Low risk | Low risk |
| Fonseca et al. | 2005 | High risk | Low risk | Low risk | Low risk | Low risk | Low risk | Low risk |
| Cooper et al. | 2005 | Low risk | Low risk | Low risk | Low risk | Low risk | Low risk | Low risk |
| Overton et al. | 2010 | Low risk | Low risk | Low risk | Low risk | High risk | Low risk | Low risk |
| Launay et al. | 2011 | Low risk | Low risk | Low risk | Low risk | Low risk | Low risk | Low risk |
| Chaiklang et al. | 2013 | High risk | Low risk | Low risk | Low risk | Low risk | High risk | Low risk |
| David Rey et al. | 2015 | Low risk | Low risk | Low risk | Low risk | Low risk | Low risk | Low risk |
| Cornejo-Juarez et al. | 2006 | Low risk | Low risk | Low risk | Low risk | Low risk | Low risk | Low risk |

Table S2. Quality assessment of prospective cohort study

| **Author** | **year** | **Selection** | | | | **Comparability** | **Outcome** | | |
| --- | --- | --- | --- | --- | --- | --- | --- | --- | --- |
|  |  | **Representativeness of the exposed** | **Selection of the non-exposed** | **Ascertainment of exposure** | **outcome was not present at start** | **Comparability of cohorts** | **Assessment of outcome** | **Was follow-up long enough** | **Adequacy of follow up** |
| Rey et al. | 2000 | * | * | * | * | * | * | * | * |
| Pasricha et al. | 2006 | * | - | * | * | - | * | * | * |
| Viega et al. | 2006 | * | - | * | * | ** | * | * | * |
| Ungulkraiwit et al. | 2007 | * | * | * | * | ** | * | * | * |
| Paitoonpong et al. | 2008 | * | NA | * | * | NA | * | * | * |
| Cruciani et al. | 2009 | * | NA | * | * | NA | * | * | * |
| Potsch et al. | 2010 | * | NA | * | * | NA | * | * | * |
| Potsch et al. | 2012 | * | NA | * | * | NA | * | * | * |
| Fuster et al. | 2016 | * | * | * | * | ** | * | * | * |

NA: not assessed
